# Choroidal metastasis from tubulopapillary renal cell carcinoma: a case report

**DOI:** 10.4076/1757-1626-2-6681

**Published:** 2009-06-29

**Authors:** Ibrahim Elghissassi, Hanane Inrhaoun, Nabil Ismaili, Hassan Errihani

**Affiliations:** Department of Medical Oncology, National Institute of OncologyRabatMorocco

## Abstract

Choroidal metastases from renal carcinoma are rare. Most reported cases describe a clear cell carcinoma histologic subtype. Metastatic tubulopapillary renal cell carcinoma to the choroid plexus is very exceptional.

We report the case of a 31-year-old man with a history of tubulopapillary renal cell carcinoma who presented two years later with metastatic disease to lungs and presternal soft tissue and three months after with choroidal metastasis revealed on ophtalmoscopic examination and magnetic resonance imaging.

The case is discussed in the framework of the existing literature about the clinical features, treatment, and prognosis of this very rare condition.

## Introduction

Kidney cancer accounts for approximately 2.5% of all adult cancers. Clear cell carcinoma is the most common histologic subtype followed by tubulopapillary renal cell carcinoma (TPRCC) who represents about 10% of all renal cell tumours [1]. This tumor metastasizes most frequently to lung, liver, bone, and subcutaneous tissues. Choroidal metastasis from TPRCC is extremely rare. The present study describes a case of choroidal metastasis from a TPRCC.

## Case presentation

A 31-year-old Berber Moroccan man was admitted in June 2005 for investigation of painless haematuria. His medical history was otherwise unremarkable. A suspicious left kidney mass was discovered and the patient underwent left radical nephrectomy. Pathological examination reported ‘moderately differentiated’ TPRCC. The tumour was confined to the pelvis, with no evidence of distant metastases at this stage.

**Figure 1.**
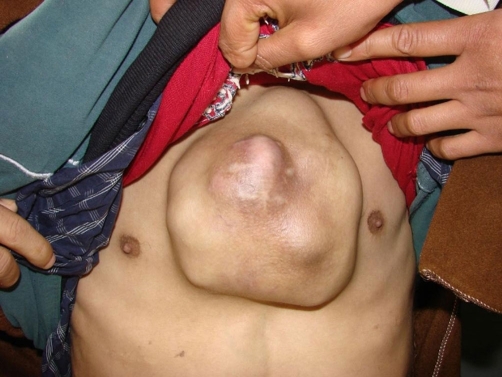
A midline anterior chest wall mass.

Two years later, he presented with a rapidly growing mass located on midline anterior chest wall (Figure 1). Thoracic CT-scan showed irregularity and sclerosis of lower part of sternum with a large presternal soft tissue mass and multiple opacities throughout both lung fields consistent with pulmonary metastases. A radionuclide bone scan revealed a single area of uptake of radiotracer in the sternum. Abdominopelvic CT-scan was normal. Routine hematological, liver function and renal function tests were within normal ranges. A biopsy of the mass was performed and histopathological examination confirmed the diagnosis of metastatic TPRCC.

**Figure 2.**
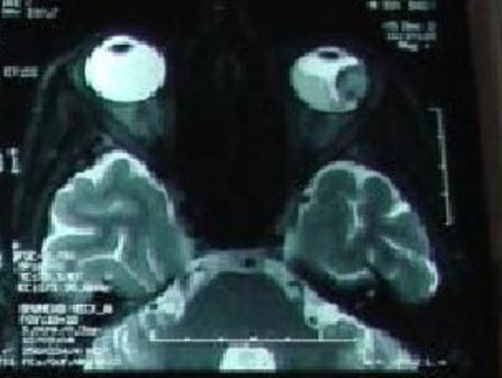
A left lateral choroidal mass with secondary retinal detachment on magnetic resonance imaging.

The patient received medical treatment which included interferon alfa-2a (9 MIU subcutaneously three times weekly) and bevacizumab (10 mg/kg every 2 weeks). After three months of this treatment, a repeat thoracic and abodminopelvic CT scan demonstrated a progressive disease with increasing of the presternal mass by 40% and appearance of multiple liver metastases. Furthermore, the patient developed severe headaches and blurred vision in the left eye. Ophthalmoscopic examination of the left eye showed a yellow choroidal mass, measuring about half a disc diameter and located below and lateral to the macular area, which was highly characteristic of choroidal metastasis. Cerebral magnetic resonance imaging was performed and showed a left lateral choroidal mass with secondary retinal detachment. This radiological aspect was consistent with the diagnosis of choroidal metastasis (Figure 2). Radiation therapy was begun to the left eye, but the patient’s condition deteriorated rapidly and he died two weeks later.

## Discussion

The first choroidal metastasis case was described by Perl in 1872 [2]. Godtfredsen, reported a 0.07% incidence of clinically detected choroidal metastasis in patients with systemic malignancy in 1944 [3]. Since then, the reported incidence of choroidal metastasis has been increasing related to advances in ophthalmic diagnostic techniques and the treatment of systemic cancers [4].

Although breast and lung cancers represent the most common source of choroidal metastases, malignancies of the gastrointestinal tract, kidney, prostate and skin have all been reported to metastasize to the choroid [5]. Most reported cases of metastasis to the choroid plexus from renal cell carcinoma describe a clear cell carcinoma histologic subtype. The mechanism of this association is still unclear. Choroidal metastasis from TPRCC, as in our case, remains very exceptional [6].

Clinically, the most common symptoms are blurring of vision, photopsia, floaters and pain whereas some patients may be asymptomatic. Typical ophtalmoscopic features include one or multiples creamy yellow choroidal lesions associated in some advanced cases with secondary retinal detachment [7].

In patients with metastatic cancer to the choroids, the most appropriate treatment seems to be a course of external beam radiation therapy. Radiotherapy can improve vision with a high response rate (63-89%) [8] and prevent pain and secondary glaucoma [9]. Systemic chemotherapy or hormonotherapy may be effective to controle choroidal metastasis with 26-81% tumor control rate reported [10,11]. In cases of painful blind eye, enucleation may be considered [12].

The prognosis for choroidal metasatsis is generally poor. The median survival was found to be as short as 8 months after the onset of metastatic disease [13].

Choroidal metastasis from renal carcinoma is a rare condition that should not be overlooked. A high index of suspicion and adequate investigation of patients with visual complaints and history of renal carcinoma are needed.

## Abbreviations

CT: Computed tomography
TPRCC: Tubulopapillary renal cell carcinoma
